# Toward stability of dynamic FC estimates in neuroimaging and electrophysiology: Solutions and limits

**DOI:** 10.1162/netn_a_00331

**Published:** 2023-12-22

**Authors:** Sonsoles Alonso, Diego Vidaurre

**Affiliations:** Department of Clinical Medicine, Center of Functionally Integrative Neuroscience, Aarhus University, Aarhus, Denmark

**Keywords:** Estimation noise, Hidden Markov model, Best ranked HMM, Hierarchical-clustered HMM, Time-varying FC, Replicability, Reproducibility

## Abstract

Time-varying functional connectivity (FC) methods are used to map the spatiotemporal organization of brain activity. However, their estimation can be unstable, in the sense that different runs of the inference may yield different solutions. But to draw meaningful relations to behavior, estimates must be robust and reproducible. Here, we propose two solutions using the hidden Markov model (HMM) as a descriptive model of time-varying FC. The first, best ranked HMM, involves running the inference multiple times and selecting the best model based on a quantitative measure combining fitness and model complexity. The second, hierarchical-clustered HMM, generates stable cluster state time series by applying hierarchical clustering to the state time series obtained from multiple runs. Experimental results on fMRI and magnetoencephalography data demonstrate that these approaches substantially improve the stability of time-varying FC estimations. Overall, hierarchical-clustered HMM is preferred when the inference variability is high, while the best ranked HMM performs better otherwise.

## Author Summary

Functional connectivity (FC) is a widely used metric in neuroimaging and electrophysiology. Characterizing the temporal evolution of FC might help to better understand the multiregion, dynamic organization of the brain that subserves human cognition. Unfortunately, most current methods for assessing time-varying FC often lack reproducibility due to estimation noise, resulting in inconsistent results. In this study, we introduce two solutions based on the hidden Markov model to enhance the stability of time-varying FC estimations. These approaches represent an important step toward improving the reproducibility of FC analysis.

## INTRODUCTION

How are different regions brought together into functional networks, and how are these dynamically organized at different spatial and temporal scales? These are important questions toward an understanding of the brain’s functional architecture (Laughlin & Sejnowski, 2003). One of the most widely used metrics to map these functional relationships is functional connectivity (FC), a measure of the statistical dependencies between pairs of brain regions (Friston, 1994). More recently, the exploration of the temporal properties of these interdependencies has revealed meaningful within-session fluctuations in FC, both from functional magnetic resonance imaging (fMRI; Fornito & Bullmore, 2010; Karapanagiotidis et al., 2020; Liégeois et al., 2019; Lurie et al., 2020; Vidaurre et al., 2017, 2018, 2021; Xie et al., 2018) and electrophysiology (Baker et al., 2014; de Pasquale et al., 2010, 2012; Núñez et al., 2021; Tewarie et al., 2019; Vidaurre et al., 2018).

Unfortunately, comparing time-varying FC results across studies poses challenges due to the variety of analytical tools available, each with their own limitations (Dafflon et al., 2022). One method for estimating time-varying FC is the hidden Markov model (HMM; Vidaurre et al., 2017) which depends on an optimization process to estimate the model parameters. As it happens with other approaches like independent component analysis (ICA; Beckmann & Smith, 2004), K-means clustering (Cabral et al., 2017), and nonnegative matrix factorization (Tewarie et al., 2019), there is a stochastic factor in the optimization algorithm that induces statistical noise in the analysis pipeline. That is, different runs of the algorithm may produce different results even on the same data. This instability in time-varying FC estimation can be influenced by factors such as the amount of data and the complexity of the model (Vidaurre et al., 2019). But to draw meaningful relations to behavior, we need to develop estimation methods that are robust and reproducible.

To address this issue, we propose two approaches to enhance the stability of time-varying FC estimation. While we focus on the HMM, analogous solutions could similarly be devised for other methods. The first approach, best ranked HMM (BR-HMM), consists of running the model several times and taking the run that best scores with respect to a given quantitative measure of model performance. In the case of the HMM, the natural choice is the free energy, which, based on Bayesian principles, represents a trade-off between fitness and model complexity. Previous studies have used the strategy of minimizing free energy to select the best model, typically considering only 10 to 20 runs (Fauchon et al., 2022; Quinn et al., 2018; Roshchupkina et al., 2022). Here, we show that, depending on the characteristics of the data, a larger number of runs might be needed to find a robust and reproducible model. The second approach, hierarchical-clustered HMM (HC-HMM), involves using subsequent hierarchical clustering to group the state time series obtained from multiple HMM runs based on their similarities. The states within each cluster are then aggregated to create a more robust representation of time-varying FC. We show the effectiveness of these methods on two separate fMRI and magnetoencephalography (MEG) datasets. Our results demonstrate that HC-HMM is computationally more efficient than BR-HMM when the variability in the model inference is high. However, when the variability is low, BR-HMM outperforms HC-HMM.

## MATERIALS AND METHODS

### Data Description

#### Resting-state fMRI Human Connectome Project data.

We used publicly available fMRI data from 100 subjects from the Human Connectome Project (HCP; Van Essen et al., 2013). For each participant, we considered data from four resting-state sessions of approximately 15 min each. Please refer to Van Essen et al. (2012) for full details about the acquisition and preprocessing of the data. In brief, 3T whole-brain fMRI data were acquired with a spatial resolution of 2 × 2 × 2 mm and a temporal resolution of 0.72 s. All fMRI processing was performed using FSL (Jenkinson et al., 2012) including minimal high-pass temporal filtering (>2,000 s FWHM) to remove the linear trends of the data, and artifact removal using ICA + FIX (Griffanti et al., 2014). No low-pass temporal filtering or global signal regression was applied. Group spatial ICA was performed using MELODIC (Beckmann & Smith, 2004) to obtain a parcellation of 25 independent components (ICs). These time series were directly obtained from the HCP ‘PTN’ Parcellation + Timeseries + Netmats (specifically, the first 100 subjects from the 812 ‘recon2’ subjects’ version). Whereas the HCP provides a higher number of IC parcellations, the 25-ICA parcellation was sufficient to map the dynamics of FC as it covers the major functional networks while keeping the computational cost low. The employed dataset matrix for this analysis consists of rows equivalent to the number of time points per session and subject (1,200 × 4 × 100) and 25 columns corresponding to the number of IC components.

#### Resting-state MEG UK-MEG data.

The MEG dataset consisted of 5-min resting-state recordings from 10 subjects obtained from the UK-MEG Partnership (Hunt et al., 2016), which recruited 77 healthy participants at the University of Nottingham. Resting-state MEG data were acquired using a 275-channel CTF MEG system (MISL, Coquitlam, Canada) operating in third-order synthetic gradiometry configuration, at a sampling frequency of 1200 Hz. For MEG coregistration, MRI data collected with a Phillips Achieva 7T was employed. MEG data were downsampled to 250 Hz using an anti-aliasing filter, filtered out frequencies <1 Hz, and source-reconstructed to 42 dipoles (covering whole-brain cortical but not subcortical regions) using a linearly constrained minimum variance scalar beamformer. Out of these dipoles, 38 were created through ICA decomposition on resting-state fMRI data from the HCP, which was previously used to estimate large-scale static FC networks in MEG (Colclough et al., 2016). The remaining dipoles relate to the anterior and posterior precuneus. A weighted mask was employed to project the data to brain space, with each area having its highest value near the center of gravity. Bad segments were manually eliminated, and spatial leakage was corrected using the technique described in Colclough et al. (2015). Twenty-two subjects were excluded due to excessive head motion or artifacts. From the remaining 55 participants (mean age 26.5 years, maximum age 48 years, minimum age 18 years, 35 men), two subsets of 10 subjects (1 to 10 for the main analysis and 11 to 20 for the validation analysis) were arbitrarily selected. This relatively low number was chosen to reduce the computational cost of our analysis, which involved several repetitions of the models’ training in order to assess their performance. The full dataset, as referenced in Vidaurre et al. (2018), can be accessed online. The dataset used for the main analysis consists of a matrix with 725,006 rows, representing the time points across subjects’ sessions, and 42 columns, corresponding to the brain regions.

### Analysis Pipeline

We applied the following methods on each dataset: (i) HMM, (ii) BR-HMM, and (iii) HC-HMM. The results of the fMRI and the MEG datasets are presented in the same order.

#### Hidden Markov model.

**Figure 1.**
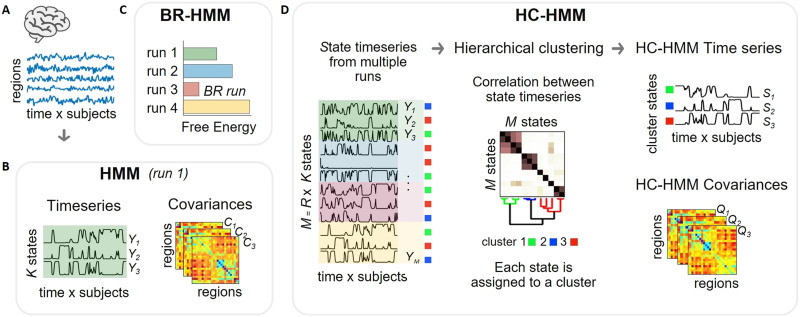
Graphical summary of the two approaches (BR-HMM and HC-HMM) to achieve reliable patterns of time-varying FC. (A) fMRI or MEG time series, temporally concatenated across subjects. (B) An HMM with *K* states was inferred from the concatenated brain time series to obtain patterns of time-varying FC (i.e., covariance matrices across regions, *C*). For the MEG dataset, the HMM was run on power time series. The state time series (*Y*) contain the probability of a given state being active at each time point. (C) The BR-HMM approach consists of running the model inference multiple times, each starting from a different random initialization, and selecting the best ranked HMM run according to the free energy, a quantitative measure that weighs the fitness of the data and simplicity of the model. (D) The HC-HMM approach involves running the HMM multiple times (*R*) and clustering the resulting state time series (*M* = *R* × *K*) according to their proximity (by means of Pearson’s correlation) to obtain more stable time-varying FC estimates. Ward’s hierarchical clustering algorithm is then applied to the *M* × *M* between-state correlation matrix to find similar state clusters. By averaging the original HMM state time series within each cluster, a set of cluster state time series are produced (denoted as *S*_*i*_). The covariances of each cluster state (denoted as *Q*_*i*_) are calculated by computing the weighted average of the covariance matrices of the original states within each cluster (*C*_*j*_), where the weights *β*_*j*_ are given by the average probability of the state activation throughout the dataset (i.e., the fractional occupancy). BR-HMM: best ranked HMM; HC-HMM: Hierarchical-clustered HMM; HMM: hidden Markov model(ling).

The hidden Markov model (HMM) is a statistical, generative model used to analyze time series, or most generally, sequential data. In the context of brain data (Figure 1A), the HMM offers a representation of neural activity as a sequence of *K* hidden states, where each state is a probability distribution, in such a way that each time point is assigned to one of the *K* states probabilistically, that is, per time point, there are *K* probabilities. As such, it can be used as a dimensionality reduction technique that maps data (time by channels or regions) to state time series (time by states). For example, the states can be represented as Gaussian distributions with the mean parameter pinned to zero and a full covariance matrix representing pairwise covariation across regions (Figure 1B), which is equivalent to a Wishart distribution (Vidaurre et al., 2021). The HMM includes a transition probability matrix indicating the probability of switching from every state to another. Mathematically, the model can be expressed as:

$$\;X_{t}|S_{t}=k∼N\left(\mu_{k},\Sigma_{k}\right)$$ (1)

where *X*_*t*_ represents the observed data at time *t*, *S*_*t*_ represents the state at time *t*, *k* is the index of the state, *N* denotes the Gaussian distribution, *μ*_*k*_ = 0 in this case, and $\sum_{k}$ is the covariance matrix for state *k*.

The hidden state variables in our model follow Markov dynamics, which implies that the probability *P*(*S*_*t*_ = *k*) is independent of the state variable’s history, given *S*_*t*−1_. Therefore, we have:

$$P\left(S_{t}=k_{1}|S_{t-1}=k_{2}\right)=\theta_{k_{1}k_{2}}\;\;\text{if}\;t>0,\;\;\;P\left(S_{0}=k\right)=\eta_{k}$$ (2)

where *θ*_*k*_1_*k*_2__ and *η*_*k*_ are the model parameters that need to be inferred, and *t* = 0 refers to the first time point of each session.

The HMM is trained at the group level to the concatenated time series across subjects, taking into account discontinuities in the data due to different sessions not being consecutive or the removal of bad segments as artifacts; this way, the first time point after a discontinuity is not modeled using the transition probability matrix, but the initial probabilities *η*. Note that, although trained at the group level, the temporal characteristics of each state (i.e., the state time series) are specific to each subject. To estimate the parameters of the HMM, we use optimization based on variational inference (Vidaurre et al., 2016), based on the minimization of a quantity called the free energy, which commences from a random initialization point. This means that the final model parameters and results can be sensitive to the initial conditions and can vary from one run to another. This sensitivity to the initial conditions is a source of statistical noise in HMMs that can lead to less stable results. To evaluate how much the results across runs differed from each other, we run the model multiple times and compared the similarity of the estimates across runs on both fMRI and MEG data. After finding the optimal state alignment by using the Hungarian algorithm (Munkres, 1957; Vidaurre, 2021), which pairs the states between runs by minimizing a cost matrix based on state dissimilarity, the similarity between two runs was measured as:

$$\text{similarity}\left(i,j\right)=1-\left(\text{cost}|\max\text{cost}\right)$$ (3)

Here, *cost* represents the dissimilarity between the paired states of run *i* and run *j*, and max *cost* is the maximum possible cost. The *similarity* ranges between 0 and 1, where a value closer to 1 indicates higher similarity between the runs.

#### Best ranked hidden Markov model.

In the BR-HMM approach (Figure 1C), we run the model multiple times and choose the run with the lowest free energy. The free energy represents a trade-off between model fitness to the data and complexity (given by how the posteriors differ from the priors). The mathematical formulas for calculating free energy in relation to HMM can be found elsewhere (Vidaurre et al., 2016). To determine the minimum number of HMM runs required for the BR-HMM approach to producing stable results, we varied the number of HMM runs (*R*) from 5 to 500 and performed eight BR-HMM repetitions for each value of *R*. That is, in each repetition, the HMM was run *R* times, and the run with the lowest free energy was chosen as the BR-HMM run. We then assessed the correspondence across estimates by measuring the similarity between each pair of BR-HMM runs.

#### Hierarchical-clustered hidden Markov model.

The HC-HMM approach (Figure 1D) uses hierarchical clustering on the state time series from multiple runs of an HMM, reducing *K*-by-*R* state time series into a reduced set of clusters, each with a cluster time series. Since the clusters tend to include states that occur consistently across runs, the generated clusters produce state time series that are more stable than the original state time series, resulting in a more reliable representation of the underlying patterns of time-varying FC. Specifically, the hierarchical clustering algorithm was fed the between-state correlation matrix *P*, containing Pearson’s correlation between every pair of state time series. The total number of states is *M* = *K* × *R*. As a distance measure, we used *D*_*ij*_ = 1 − *P*_*ij*_, where *P*_*ij*_ represents the correlation coefficient between the *i*^th^ and *j*^th^ state. The clustering algorithm starts by regarding each element as a separate cluster; then the closest pair of clusters are iteratively combined into a larger cluster. To measure the distance between two clusters, we used Ward’s linkage, which minimizes the variance of the clusters being merged. As a result, highly correlated states will be clustered together. A dendrogram was obtained from this procedure; each of the clusters can be seen as a group of states with the same underlying FC pattern. We set the number of clusters to match the number of states (*K*) in the HMM. For each cluster *i* = 1 … *K*, an aggregated state time series (denoted as *S*_*i*_) was obtained by averaging the original state time series within that cluster:

$$S_{i}=\frac{1}{N}\sum_{j=1}^{N_{i}}\left(Y_{j}\right)$$ (4)

Here, *j* = 1 … *N*_*i*_, where *N*_*i*_ represents the number of original states within the i^th^ cluster. To ensure that the cluster time series of the HC-HMM can be interpreted as probabilities, we rescaled them such that the sum across HC-HMM clusters is equal to 1.0 at each time point. To estimate the spatial patterns of time-varying FC associated with each cluster state time series, we computed one covariance matrix for each cluster (denoted as *Q*_*i*_) by taking the weighted average of the covariance matrices (*C*) of the original states within that cluster:

$$Q_{i}=\frac{1}{N}\sum_{j=1}^{N_{i}}\left(\beta_{j}C_{j}\right)$$ (5)

where *β*_*j*_ is the fractional occupancy of each original state, representing the average probability of a state activation throughout the dataset.

To evaluate the stability of the results, the process was repeated eight times and the similarity of the results across HC-HMM repetitions were compared. We also quantified the relationship between HC-HMM stability and the number of HMM runs, for various choices of *R* (from 5 to 300).

### Computational Resources and Environment

The computations were performed using MATLAB version 9.10 (R2021a) on an AMD Ryzen 9 3900X 12-Core CPU running the Linux 5.15.0-52-generic operating system. The system had 52 GB of RAM available. A single run of the HMM on the HCP dataset took a total of 477.44 s (∼7.96 min) to complete. For the MEG dataset, one run took 118.60 s (∼1.98 min) to complete.

## RESULTS

The following results, for both fMRI and MEG, follow the analysis pipelines detailed in the *Materials and Methods* section. All models were run at the group level, and all results are group-level results.

### FMRI Data

The fMRI dataset considered here has four sessions of resting-state fMRI data from 100 subjects from the HCP (Van Essen et al., 2013). The data were projected onto 25 ICs, which we used as input time series. Whole-brain patterns of time-varying FC were obtained by applying an HMM to the concatenated (standardized) time series for all subjects (25 ICs by [time × sessions × subjects]). The HMM was modeled with *K* = 12 states, as in Vidaurre et al. (2017); here each state represents a reoccurring pattern of FC. It is important to note that the choice of *K* is not based on ground truths but on practical reasons (Vidaurre, 2023), so that different numbers of states can be used to represent patterns of FC in different levels of detail.

#### HMM stability.

**Figure 2.**
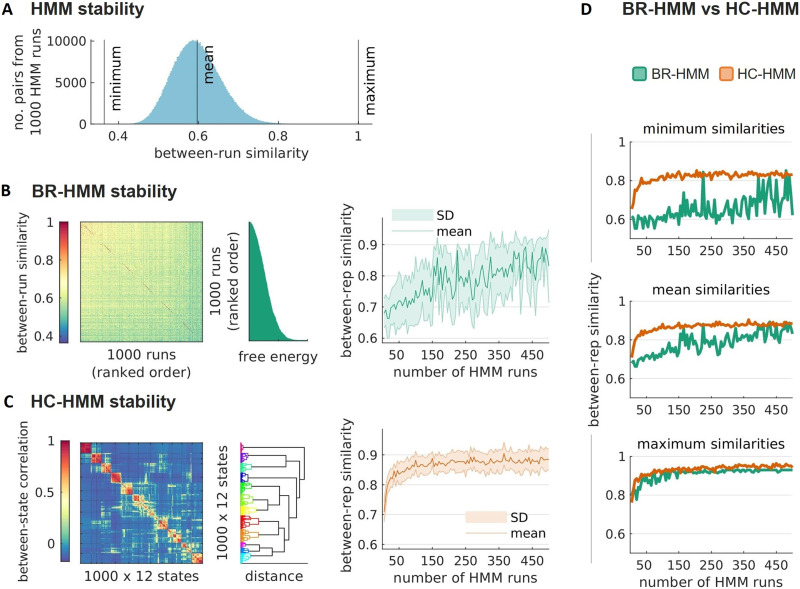
Stable time-varying FC estimations from resting-state fMRI data. (A) Histogram of between-run similarities computed from 1,000 HMM runs (*N* = 499,500 pairs of runs). The similarity of each pair of HMM runs was computed as the sum of the joint probabilities across two sets of *K* = 12 state time series (see *Materials and Methods*). (B, left to right) Run-by-run matrix of between-run similarities, with the runs sorted in ascending order based on their free energy; free energy levels for each HMM run sorted in ascending order; between-repetition similarities of the BR-HMM approach as a function of the number of HMM runs *R*, from 5 to 500, in steps of 5. (C, left to right) Matrix of Pearson’s correlation coefficients between pairs of state time series with states ordered according to hierarchical clustering (*K* clusters, the total number of states is *R* × *K* = 12,000); dendrogram showing the states within each cluster; between-repetition similarities of the HC-HMM approach as a function of *R* (from 5 to 500, in steps of 5). (D, top to bottom) Overlay plots of the minimum, mean and maximum similarity scores across repetitions as a function of *R* (BR-HMMs in green, and HC-HMMs in orange).

The stability of the HMM inference was evaluated using the between-run similarities across 1,000 runs. The similarity between two runs was computed as the sum of the joint probabilities between two sets of aligned state time series (see *Materials and Methods*). Each similarity score has a value between 0 and 1, which is proportional to how close the set of states of a run is to the set of states of a different run. A histogram of the similarity scores for each pair of runs revealed a minimum similarity score of 0.36, a maximum of 0.99, and an average score of 0.59 (Figure 2A). This indicates that the states inferred by the HMM are to some extent different across runs of the inference, as shown previously in Vidaurre et al. (2019).

#### BR-HMM stability.

The BR-HMM approach is based on selecting the run that is best ranked according to the free energy. How well this approach will work depends on whether the estimates generated from BR-HMM runs (i.e., the runs with the lowest free energy) represent similar HMM solutions. In Figure 2B-*left*, a matrix of similarities is presented across the estimates obtained from 1,000 HMM runs, sorted in ascending order based on their free energy values. The between-run similarity matrix reveals that the HMM runs with the lowest free energy values are very similar to each other.

To better evaluate the stability of BR-HMM runs (i.e., the HMM runs with the lowest free energy among *R* runs), we conducted eight repetitions of the BR-HMM approach. We then assessed the similarity between the results across repetitions for different numbers of HMM runs, ranging from *R* = 5 to *R* = 500. The stability of the BR-HMM estimates gradually improves as the number of runs increases, as depicted in Figure 2B-*right*. However, the standard deviation of approximately 0.08 indicates that, still, there is some variability across BR-HMM runs. While some pairs of BR-HMM runs have a similarity of 0.93, others cannot reach 0.7, suggesting that not all BR runs converge to the same underlying HMM solution. Therefore, many HMM runs (here, 500) might be needed for reaching a robust solution.

Overall, our findings suggest that the stability of BR-HMM estimates is dependent on having enough runs of the HMM to adequately describe the landscape of possible solutions. As the complexity of the model increases, more runs are needed, which can be computationally intensive.

#### HC-HMM stability.

The HC-HMM approach utilizes hierarchical clustering to group the state timeseries obtained from multiple HMM runs on a reduced set of timeseries based on their similarity. The clustering is based on a between-state similarity matrix, given by the Pearson’s correlation between the state timeseries. This method thus provides a robust and stable estimation of state timeseries by integrating information from multiple runs of the HMM.

Here, the total number of states was *R* × *K* = 1,000 × 12; the number of clusters was set to 12 to match *K* (although a different number of clusters could be used). In Figure 2C-*left*, a Pearson’s correlation matrix between each pair of state time series is presented, with the states ordered according to the clustering. Clusters of similar states tend to concentrate around the main diagonal. It is important to note that a lack of correlation between clusters is expected as the states derived from the HMM are mutually exclusive (i.e., the sum of the probabilities of activation across states at a given timepoint for a given HMM is 1). The resulting clusters are depicted in the dendrogram of Figure 2C-*middle* and were used to generate cluster state time series by averaging the original state time series within each cluster.

We observe variations in the reproducibility of states at the level of individual HMM runs as reflected in the matrix of between-state correlation. Some states exhibit higher reproducibility, indicating a higher likelihood of being consistently identified across random runs of the HMM. The stability of each cluster is reflected by the clusters’ size and color; the size of the cluster corresponds to the number of runs that converged to the same underlying state, and the color represents the level of correlation between the states of different runs. The depicted dendrogram shows the clustering structure.

To determine the minimum number of runs required for the HC-HMM approach to returning stable results, we assessed its performance across various numbers of runs, ranging from 5 to 500. To do so, we repeated the HC-HMM procedure eight times for each value of *R* and compared the similarities across the cluster state timeseries (Figure 2C-*right*). Our results showed that the HC-HMM approach achieved prominent levels of stability with just 50 runs, with similarities across HC-HMM estimates exceeding 0.84. In general, adding more runs did not significantly improve the stability of HC-HMM beyond 0.89 (*SD* = 0.03).

#### BR-HMM versus HC-HMM stability.

The between-repetition similarities of both approaches are also represented in Figure 2D. The minimum, mean, and maximum similarity scores of HC-HMM were consistently higher than the BR-HMM’s. The HC-HMM approach achieved stability above 0.84 with just 50 runs, while more than 500 runs were needed for BR-HMM to reach the same levels of stability.

Supporting Information Figure S1 offers further insights into the underlying FC patterns of the states derived from each approach. Additionally, it demonstrates the spatial correspondence between the states obtained from the two approaches based on 1,000 HMM runs. Notably, the spatial correspondence (measured as the Pearson’s correlation between the covariance matrices of aligned states) exceeded 0.88 in 9 out of 12 states, reflecting large consistency between the two approaches. Supporting Information Figure S2 describes the dynamic state metrics for each state, including fractional occupancies (proportion of time spent in each state), lifetimes (time spent in each state visit), and switching rates (frequency of change between states over time). While there were no large differences in the distribution of the evaluated metrics between the two approaches, there were small variations in the duration of visits for two HC-HMM states compared to their corresponding states in the BR-HMM approach.

Taken together, despite the stochastic nature of the HMM inference, these results show that dynamic changes in FC can be reliably captured in fMRI if appropriate methods are used. While the BR-HMM approach required hundreds of inference realizations to describe the landscape of possible HMM solutions, HC-HMM achieved high stability with just a fraction of the runs. This highlights the advantages of the HC-HMM approach, particularly when inference is variable enough that the number of runs needed to obtain minimal values of free energy is high.

### MEG Data

The MEG dataset utilized in this study comprised 5-min resting-state recordings obtained from a total of 10 subjects (subjects 1 to 10 of the full dataset, see *Materials and Methods*). For easier comparison with the fMRI results, we specifically examined the amplitude of the signal. We analyzed the power time series within the alpha band frequency range (8–12 Hz) as well as a wider frequency range (4–30 Hz), rather than utilizing the raw timeseries data. The HMMs were trained with *K* = 6 (following the recommendation by Baker et al. [2014] of using fewer than eight states) and *K* = 12.

#### HMM stability.

**Figure 3.**
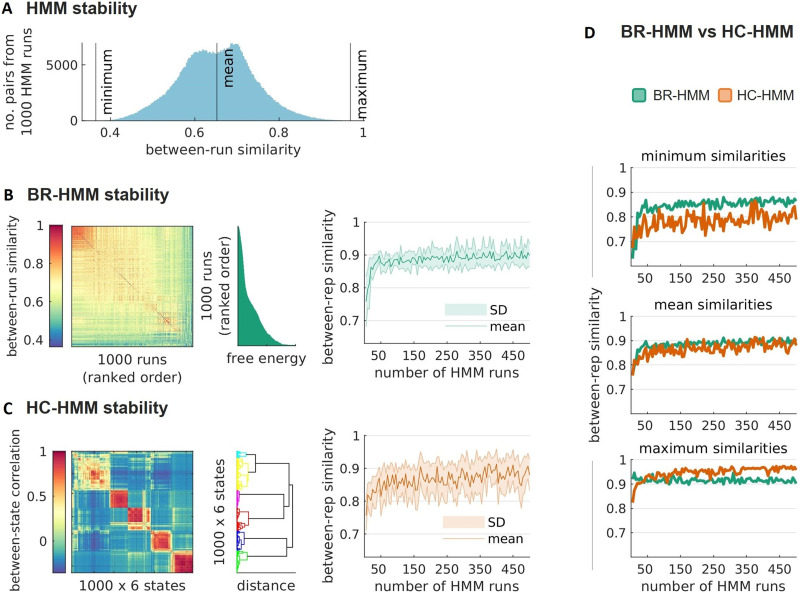
Stable time-varying FC estimations from resting-state (alpha band) MEG data. (A) Histogram of between-run similarities computed from 1,000 HMM runs (*N* = 499,500 pairs of runs). The similarity of each pair of HMM runs was computed as the sum of the joint probabilities across two sets of *K* = 6 state time series (see *Materials and Methods*). (B) (left to right) Run-by-run matrix of between-run similarities, with the runs sorted in ascending order based on their free energy; free energy levels for each HMM run sorted in ascending order; between-repetition similarities of the BR-HMM approach as a function of the number of HMM runs *R*, from 5 to 500, in steps of 5. (C) (left to right) Matrix of Pearson’s correlation coefficients between pairs of state time series with states ordered according to hierarchical clustering (*K* clusters, the total number of states is *R* × *K* = 6,000); dendrogram showing the states within each cluster; between-repetition similarities of the HC-HMM approach as a function of *R* (from 5 to 500, in steps of 5). (D) (top to bottom) Overlay plots of the minimum, mean and maximum similarity scores across repetitions as a function of *R* (BR-HMMs in green, and HC-HMMs in orange).

To evaluate the stability of the HMM inference for a model with *K* = 6 states, the model was run 1,000 times and the similarity across runs was computed as the sum of the joint probabilities under optimal state alignment (using the Hungarian algorithm, see *Materials and Methods*). Figure 3A displays a histogram of the similarities across runs, which shows a minimum similarity score of 0.37, a maximum of 0.97, and an average score of 0.65 (*SD* = 0.09). Increasing the number of states to *K* = 12 resulted in a substantial increase in inference variability, as illustrated in Supporting Information Figure S3. These results underscore the importance of *K* in the reliability of the results.

#### BR-HMM stability.

Like in the fMRI dataset, the BR-HMM approach was used to select the best run among multiple HMM runs based on the lowest free energy value. Figure 3B, left, shows the free energy values for the 1,000 HMM runs sorted in ascending order, with highly stable runs having the lowest free energy values, as evidenced by the high similarity scores among these runs. The relationship between free energy and stability in this dataset was clearer than in the fMRI dataset. To determine the number of HMM runs needed to reach a stable minimal value of free energy, the stability of estimates obtained from BR-HMM runs was evaluated through eight repetitions of the BR-HMM method. The similarity of results across repetitions was compared for different numbers of HMM runs, ranging from *R* = 5 to *R* = 500. As the number of HMM runs increased, the stability of estimates obtained from BR-HMM runs improved. Figure 3B, right, illustrates that a stable mean similarity of 0.87 and a standard deviation of 0.06 were achieved at approximately 50 runs. Adding more runs did not lead to significant improvements in stability beyond this point.

#### HC-HMM stability.

After running multiple HMM runs, we used the HC-HMM approach to group state time series based on their similarity. As before, the clustering was performed on a between-state similarity matrix, where similarity was measured as the Pearson’s correlation between each pair of state time series. The correlation coefficients between each pair of state time series are shown in Figure 3C (left), with the states ordered based on the hierarchical clustering. The resulting clusters were depicted in a dendrogram; cluster state time series were generated by averaging the original state time series within each cluster. To assess the stability of the HC-HMM approach, we repeated the procedure eight times for each value of *R* and compared the similarities across the cluster state time series for various numbers of runs (5 to 500). The results showed that the HC-HMM approach consistently yielded higher similarities between cluster states obtained from the original HMM states over as few as 20 runs (Figure 3C, right).

#### BR-HMM versus HC-HMM stability.

Here, the stability obtained using the BR-HMM and HC-HMM were in general similar (Figure 3D). While the mean stability of BR-HMM is only slightly better than HC-HMM, the latter shows a wider standard deviation. Therefore, although the estimations across repetitions of the HC-HMM approach may reach values close to one, or even higher than those of the BR-HMM approach, this was not always the case.

Supporting Information Figure S4 presents brain maps depicting the FC states obtained from both the BR-HMM and HC-HMM approach, acquired from 1,000 HMM runs. The figure also illustrates the spatial correspondence between the states derived from the two approaches, measured as the Pearson’s correlation between the covariance matrices of aligned states. The results reveal a mean correspondence of 0.93 (*SD* = 0.1), indicating a substantial agreement between the approaches. Notably, four out of the six states exhibited a nearly perfect correspondence of 1. As shown in Supporting Information Figure S5, we assessed the temporal characteristics of the states, including metrics such as fractional occupancy, lifetime, and switching rate. We compared these metrics across the BR-HMM and HC-HMM approaches. Our analysis showed that there were no larger differences in the distribution of the evaluated metrics between the two approaches. The most noticeable discrepancies were observed in the fractional occupancy of the two states that most differed spatially, and in the lifetime of one of those states.

To validate these conclusions, we performed the analysis on a separate group of subjects (subjects 11 to 20 of the full dataset) and obtained consistent results, as shown in Supporting Information Figure S6. A comparative analysis of the FC states across the two datasets demonstrated a nearly one-to-one correspondence for the BR-HMM approach in all states. The HC-HMM approach exhibited high correspondence in most states, except for one, where the correspondence was negligible, illustrating the influence of the added complexity of HC-HMM on its performance.

Last, we extended our analysis to consider a broader frequency range of 4–30 Hz. The results, presented in Supporting Information Figure S7, provide clear evidence that both approaches significantly improved the stability of HMM estimations, even with a limited number of runs (50 runs). Both approaches achieved high-performance levels with mean stability exceeding 0.9. However, in this case, the HC-HMM approach displayed slightly higher stability compared to the BR-HMM approach. This difference could be attributed to the increased between-run variability observed when considering a wider frequency range as opposed to the narrower alpha band.

Taken together, our results demonstrate that both approaches can improve the stability of time-varying FC patterns in MEG data. However, when the lowest free energy runs exhibit variations within the same HMM decomposition, as opposed to representing alternative HMM representations, the simpler BR-HMM approach appears sufficient to capture the underlying dynamics, while the additional complexity introduced by HC-HMM may hinder its performance.

## DISCUSSION

Many studies in network neuroscience use sliding windows to estimate time-varying FC. The sliding window approach involves dividing the time series data into smaller time windows and estimating the FC for each window. By sliding the window across the time series, the FC can be estimated as a function of time. However, this approach is prone to statistical variability: estimates for different windows might be spuriously different just because there is not enough data in the window to yield a stable estimation. Alternatively, the HMM characterizes time-varying FC using a finite number of states (Vidaurre et al., 2017), in such a way that states have much more data available to be estimated. Unlike the sliding windows approach, and like methods based on instantaneous FC (Tewarie et al., 2019), the HMM allow modeling the FC variability on a timepoint-by-timepoint basis. However, similar to other models, the estimation of model parameters in the HMM also relies on an optimization procedure. This process is stochastic in nature, and coupled with the inherent noise in the data, different runs of the inference algorithm can yield different estimates of the model parameters, including different estimates of time-varying FC. This stochasticity in the inference poses an interpretation challenge, as different runs of the inference algorithm can produce different results. This, in turn, hampers the reproducibility of the findings, ultimately impacting our ability to accurately predict outcomes or establish meaningful relationships with behavior.

In our study, we proposed two approaches, BR-HMM and HC-HMM, to improve the stability of HMM estimates. The BR-HMM approach selects the best estimate of the time-varying FC by minimizing the free energy. While running the HMM multiple times can improve performance and reduce variability, the computational cost of the BR-HMM approach, however, can be a limiting factor, particularly when the number of runs needed to obtain minimal values of free energy is high. This is especially true for the more complex models, which may require more runs to explore the space of possible solutions sufficiently. In such cases, HC-HMM is recommended, as it is better suited to handle greater complexity of the data.

Some alternatives to hierarchical clustering are principal component analysis or nonnegative matrix factorization. However, unlike the HC-HMM approach, these would produce components that do not follow the assumptions of the HMM and are therefore harder to interpret. For instance, having an HMM-compatible model structure is particularly useful for characterizing individual FC profiles in terms of their temporal characteristics, including their activation probability and duration, as well as identifying state transitions. A potential avenue for further improvement in the HC-HMM method is related to the selection of the number of clusters. In this study, we chose to match the number of clusters to the number of states in each individual HMM. However, this may not always be the most appropriate choice. A data-driven approach for determining the optimal number of clusters could potentially yield more stable results. One method would be the elbow criterion, which identifies the point where the rate of change in the sum of squares levels off. Another option would be to visually inspect the cluster assignments and exclude any states that do not clearly belong to any of the clusters, which could improve the interpretability and robustness of the results obtained from the HC-HMM at the expense of having a less automatic approach.

While the proposed methods in this work primarily focus on reducing inference variability arising from the randomness of the optimization procedure, there may be other sources of instability in the estimation process that remain unaddressed. For example, the complexity of the landscape of FC dynamics may make it difficult to accurately describe the data with the parameters of the model and this could contribute to variability in the estimation results. Alternatively, estimation noise may arise due to limitations in the amount or quality of data available or high signal-to-noise ratio probabilities (Lee et al., 2010). To enhance the accuracy and stability of the model, future research should explore strategies to mitigate variability caused by these factors. One potential approach is to investigate the impact of different model configurations alongside the proposed methods for mitigating variability. By refining the model parameters to better capture the complexity of FC dynamics, it may be possible to further reduce the variability in the estimation results and obtain more reliable and accurate estimates of time-varying FC.

Besides the HMM, there are other methods aimed at modeling the timepoint-by-timepoint variability of FC. One example involves combining instantaneous FC measures with nonnegative tensor factorization, as presented by Ponce-Alvarez et al. (2015) and Tewarie et al. (2019). These also rely on random initializations, and similar solutions could be devised. Further work incorporating diverse approaches, optimization procedures, and underlying assumptions, and, critically, taking into account their stability, can lead to a deeper understanding of the intricate landscape of brain activity.

## CONCLUSION

Reproducibility is a growing concern in neuroimaging, and this issue can be further complicated by the stochastic nature of various methods’ inference. Focusing on the HMM for characterizing time-varying FC, we propose two methods for achieving stable estimations: BR-HMM and HC-HMM. These methods greatly enhance estimation stability. However, while BR-HMM can produce higher stability scores, its computational cost may be a limiting factor in some cases. Conversely, HC-HMM provides a computationally affordable solution. By reducing the inference variability that originates from the randomness of the optimization procedure, the proposed methods can thus yield more stable and reliable estimates of time-varying FC, which can aid in our understanding of neural processes and cognitive functions.

## DATA AVAILABILITY

The fMRI data were provided by the Human Connectome Project, WU-Minn Consortium (Principal Investigators: David Van Essen and Kamil Ugurbil; 1U54MH091657) funded by the 16 NIH Institutes and Centers that support the NIH Blueprint for Neuroscience Research; and by the McDonnell Center for Systems Neuroscience at Washington University. The MEG dataset is part of a larger dataset acquired in Nottingham in the context of the MEG UK Partnership and is not currently available as it contains data from human participants including structural scans. The data are held by the MEG UK Partnership, and access to the MEG UK Database can be requested at https://meguk.ac.uk/contact. Preprocessed (and parcellated) data containing the time series as they were fed to the HMM can be accessed at https://github.com/sonsolesalonsomartinez/reproducibleHMM.

## CODE AVAILABILITY

The software comprises the open access code repositories https://github.com/OHBA-analysis/HMM-MAR and custom MATLAB scripts https://github.com/sonsolesalonsomartinez/reproducibleHMM.

## ACKNOWLEDGMENTS

For open access, the authors have applied a CC BY NC ND public copyright license to any Author Accepted Manuscript version arising from this submission.

## SUPPORTING INFORMATION

Supporting information for this article is available at https://doi.org/10.1162/netn_a_00331.

## AUTHOR CONTRIBUTIONS

Sonsoles Alonso: Conceptualization; Data curation; Formal analysis; Investigation; Methodology; Software; Validation; Visualization; Writing – original draft; Writing – review & editing. Diego Vidaurre: Conceptualization; Data curation; Formal analysis; Funding acquisition; Investigation; Methodology; Project administration; Resources; Software; Supervision; Validation; Visualization; Writing – original draft; Writing – review & editing.

## FUNDING INFORMATION

Diego Vidaurre, Novo Nordisk Fonden (https://dx.doi.org/10.13039/501100009708), Award ID: NNF19OC-0054895. Diego Vidaurre, European Research Council (https://dx.doi.org/10.13039/501100000781), Award ID: ERC-StG-2019-850404. Diego Vidaurre, Wellcome Trust (https://dx.doi.org/10.13039/100010269), Award ID: 15573/Z/19/Z.

## TECHNICAL TERMS

HMM: Hidden Markov model is a probabilistic model used to analyze sequential (time series) data, representing it as generated by a discrete number of distributions, or states.
BR-HMM: Best-ranked HMM refers to the model with the lowest free energy among multiple runs of the inference.
Free energy: Quantitative measure combining fitness and model complexity.
HC-HMM: Hierarchical-clustered HMM denotes stable aggregated state time series obtained from multiple runs using hierarchical clustering.
Hierarchical clustering: Method for grouping state time series based on their similarity, i.e., Pearson’s correlation.
Between-state correlation: Pearson’s correlation between-state time series from different HMM runs.
Between-run similarity: Sum of joint probabilities between-state time series from different HMM runs.
Between-repitition similarity: Sum of joint probabilities between-state time series from different HC-HMM repetitions.
Inference variability: Variability in time-varying FC estimations caused by the random initialization of the HMM.
