# Supplementary material for: Toward stability of dynamic FC estimates in neuroimaging and electrophysiology: Solutions and limits
Source: Netw Neurosci. 2023 Dec 22;7(4):1389–403. doi: 10.1162/netn_a_00331 (PMC10713011; doi:10.1162/netn_a_00331)
Supplement: Supplementary file 1 [file netn-7-4-1389-s001.pdf]

Supplementary Figures

corresponding to

Towards stability of dynamic FC estimates in neuroimaging and electrophysiology: solutions and limits

Sonsoles Alonso and Diego Vidaurre

a) HMM states from BR-HMM

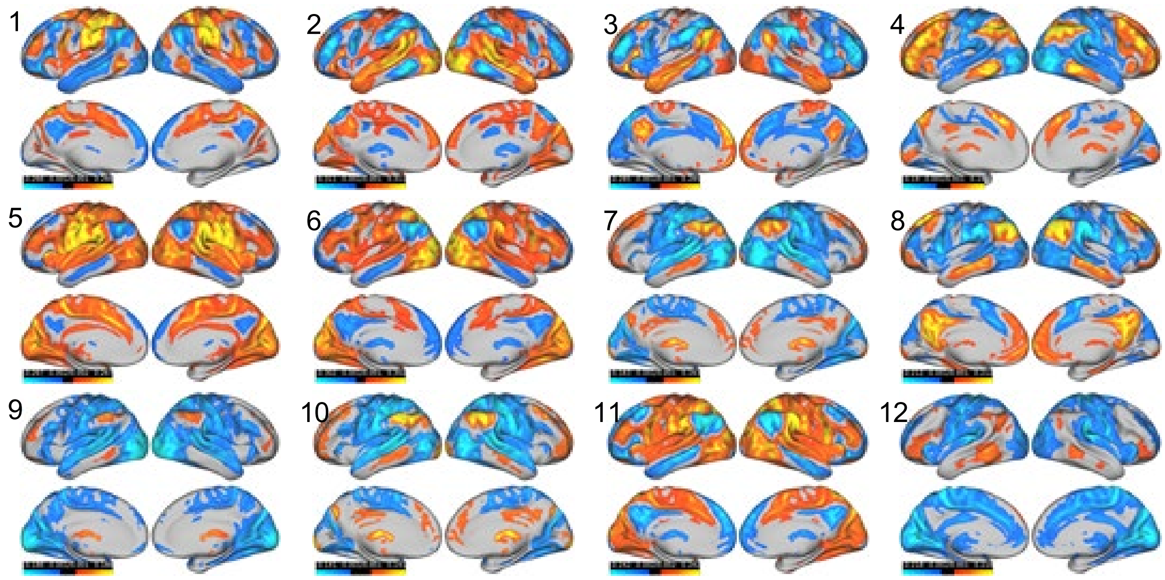

b) Aggregated HMM states from HC-HMM

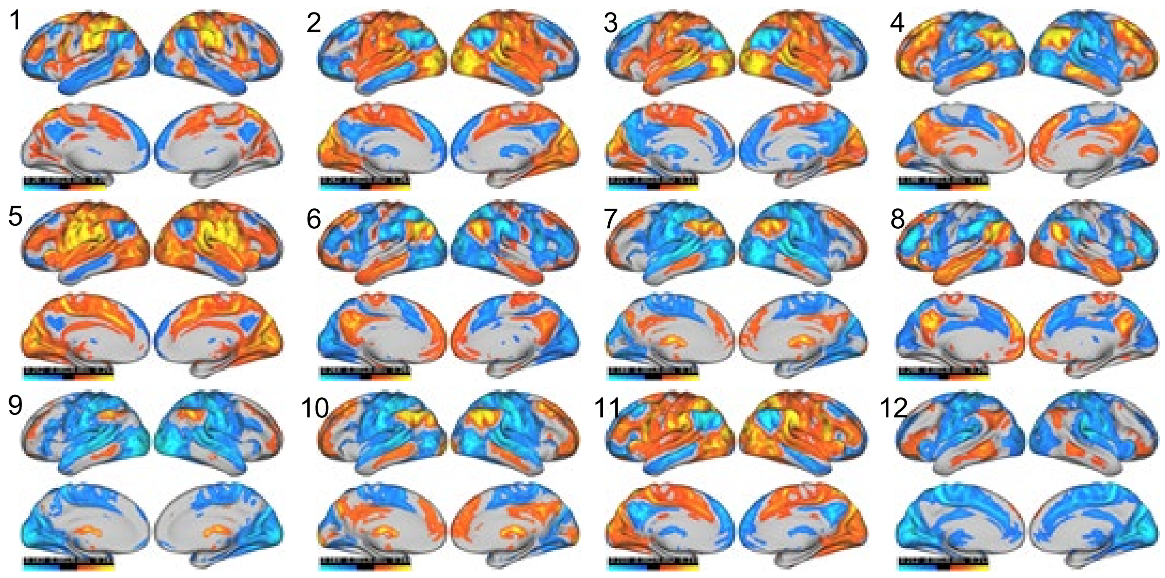

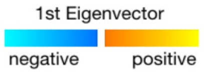

c) Correspondence between HC-HMM and BR-HMM

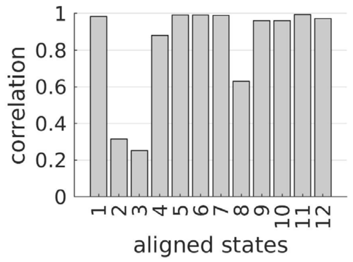

Supplementary Figure 1. Spatial representation of resting-state fMRI FC states. a) Brain connectivity maps of fMRI FC states obtained from BR-HMM. **b)** Brain connectivity maps of fMRI FC states obtained from HC-HMM. Brain connectivity maps were created using the 1st Eigenvector of each state's covariance matrices. **c)** To calculate the between-state correlation of each spatial map, we aligned the BR-HMM states to the HC-HMM states, preserving the order of the state clusters shown in **Figure 2c**'s dendrogram. We then computed the Pearson correlation between the covariance matrices for each pair of aligned states.

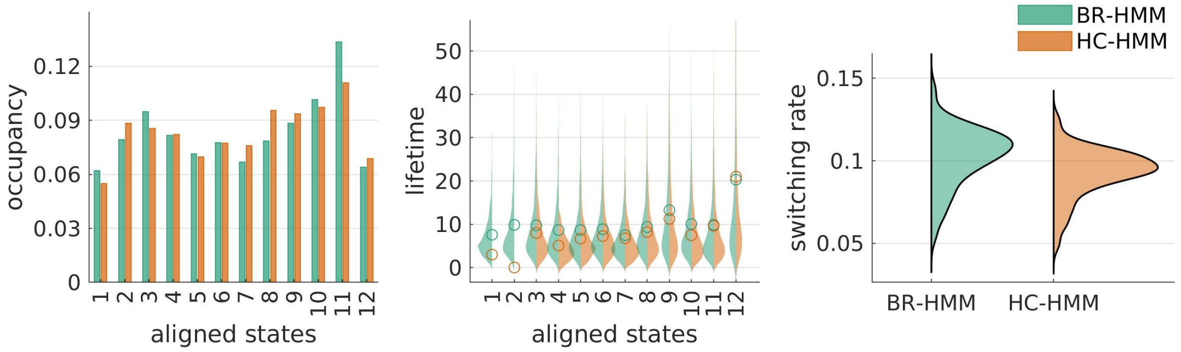

Supplementary Figure 2. Temporal metrics of fMRI FC states from BR-HMM and HC-HMM. Distribution of state fractional occupancies (proportion of time spent in each state), lifetimes (time in TRs spent in each state visit) and switching rate (frequency of change between states over time) were calculated separately for each session and state. Because the order of the states that result from an HMM is arbitrary, the BR-HMM states were first aligned to the HC-HMM states, thereby maintaining the order of the state clusters shown in the dendrogram of **Figure 2c**.

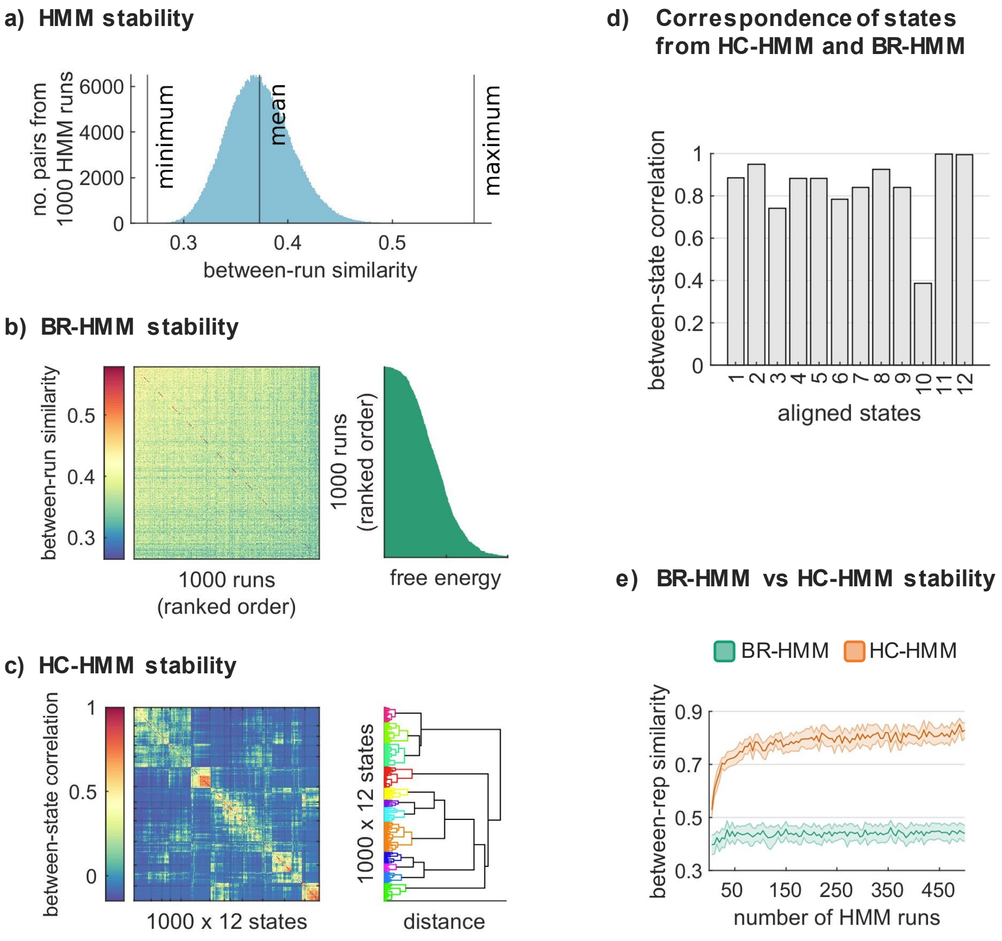

Supplementary Figure 3. Time-varying FC estimations of MEG recordings within the alpha band using K=12. a) Histogram of between-run similarities computed from 1000 HMM runs (N=499500 pairs of runs). The similarity of each pair of HMM runs was computed as the sum of the joint probabilities across two sets of K=12 state timeseries. This variability was significantly higher than the model with K=6 states illustrated in **Figure 3** of the main article, indicating that a higher number of states can increase the inference variability of HMM models. **b)** Run-by-run matrix of the between-run similarities, with the runs sorted in ascending order based on their free energy; free energy levels for each HMM run sorted in ascending order. **c)** Matrix of Pearson correlation coefficients between pairs of state timeseries with states ordered according to hierarchical clustering (K clusters, the total number of states is $R \times K$ =12000); dendrogram showing the states within each cluster. **d)** Spatial correspondence of states across approaches. The states from the BR-HMM were first aligned to the states from the HC-HMM (thereby maintaining the order of the state clusters shown in the dendrogram of panel c). Then the Pearson correlation was applied to the covariance matrix associated with each state **e)** between-repetition similarities of the BR-HMM (green) and the HC-HMM (orange), as a function of the number of HMM runs R, from 5 to 500, in steps of 5.

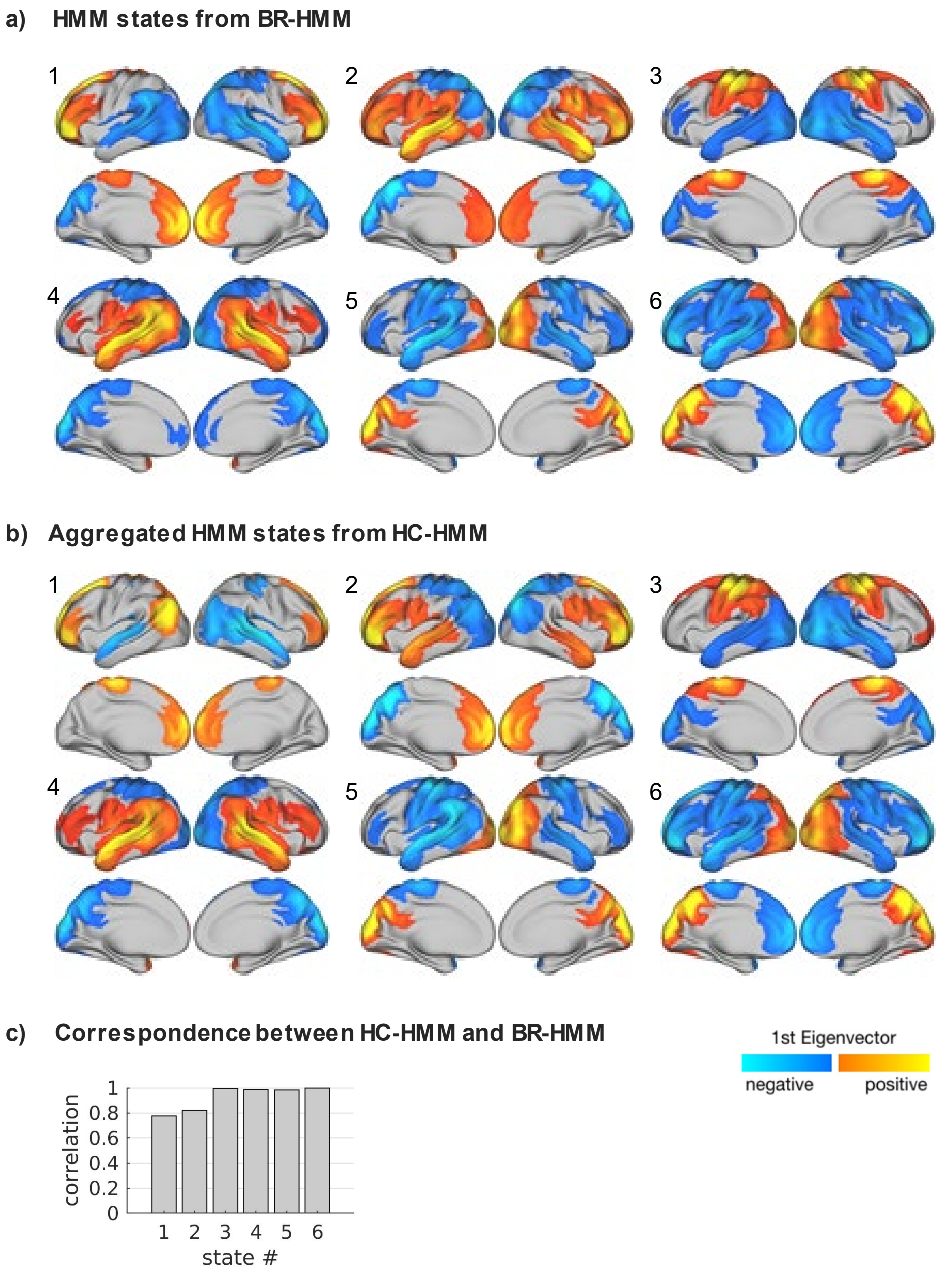

Supplementary Figure 4. Spatial representation of alpha band resting-state MEG FC states. a) Brain connectivity maps of Alpha band MEG FC states obtained from BR-HMM. **b)** Brain connectivity maps of Alpha band MEG FC states obtained from HC-HMM. The maps were generated using the 1st Eigenvector of each state's covariance matrices. **c)** To assess the correspondence between states obtained from the HC-HMM and BR-HMM approaches, the between-state correlation of each spatial map was computed. This involved calculating the Pearson correlation between the covariance matrices of aligned states. To maintain the order of the state clusters shown in the dendrogram of **Figure 3c**, the BR-HMM states were aligned to the HC-HMM states.

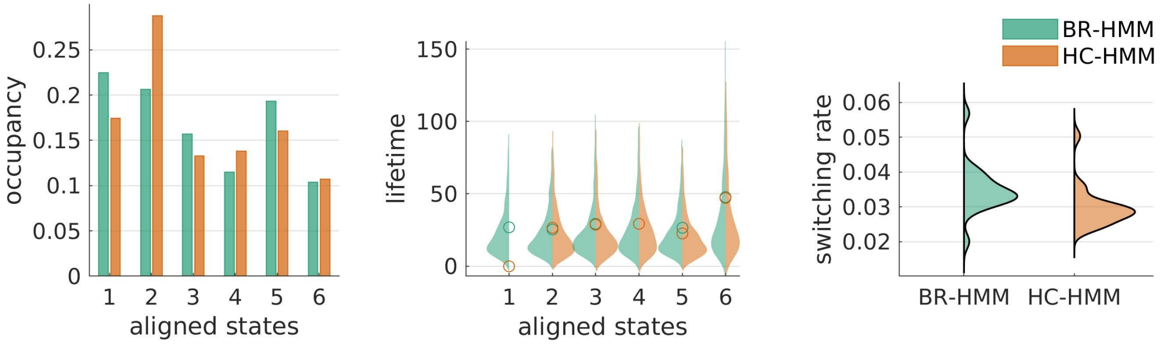

Supplementary Figure 5. Temporal metrics of alpha band resting-state MEG FC states from BR-HMM and HC-HMM. Distributions of the fractional occupancies, lifetimes and switching rates across subjects and for each state. The states from the BR-HMM approach were aligned to the states from the HC-HMM approach, preserving the order of the state clusters shown in **Figure 3c**'s dendrogram.

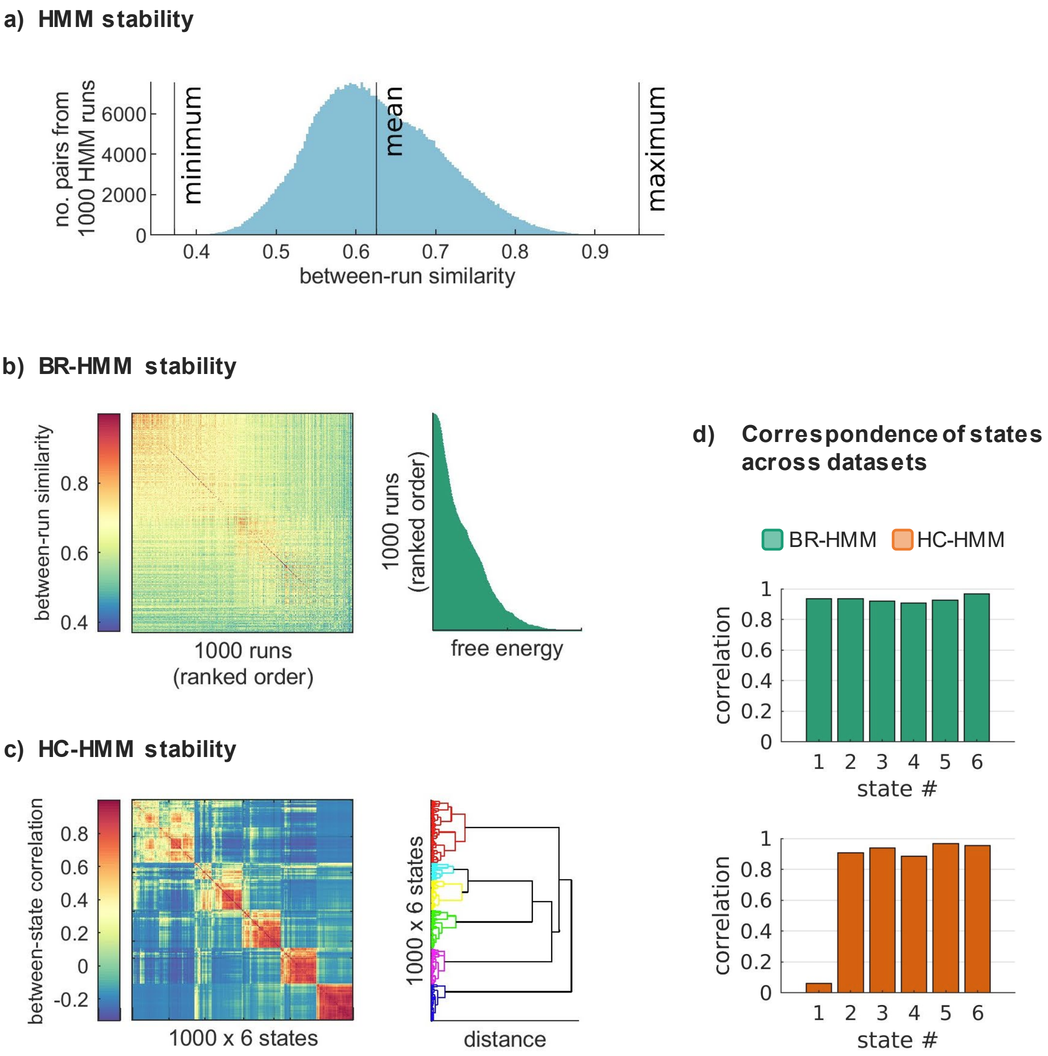

Supplementary Figure 6. Time-varying FC estimations of MEG recordings within the alpha band from the validation dataset (subjects 11 to 20). **a)** Histogram of between-run similarities computed from 1000 HMM runs (N=499500 pairs of runs). The similarity of each pair of HMM runs was computed as the sum of the joint probabilities across two sets of K=6 state timeseries. **b)** (*left* to *right*) Run-by-run matrix of the between-run similarities, with the runs sorted in ascending order based on their free energy; free energy levels for each HMM run sorted in ascending order. **c)** (*left* to *right*) Matrix of Pearson correlation coefficients between pairs of state timeseries with states ordered according to hierarchical clustering (K clusters, the total number of states is $R \times K$ =6000); dendrogram showing the states within each cluster. **d)** Correspondence between states of the validation dataset (shown in this figure) and the original dataset (shown in **Figure 3**) for both, BR-HMM (top) and BR-HMM (bottom).

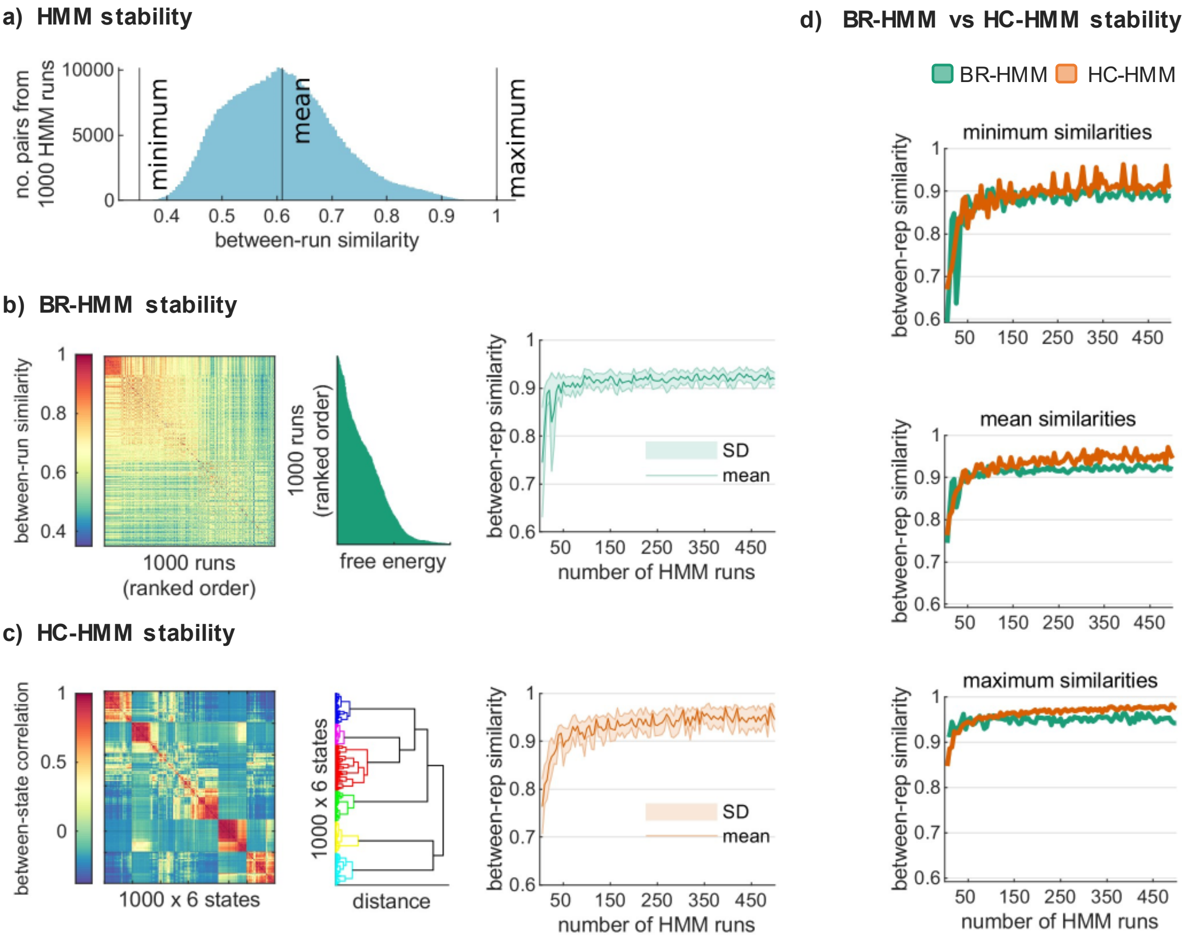

Supplementary Figure 7. Time-varying FC estimations of MEG recordings within the broadband (4-30 Hz). **a)** Histogram of between-run similarities computed from 1000 HMM runs (N=499500 pairs of runs). The similarity of each pair of HMM runs was computed as the sum of the joint probabilities across two sets of K=6 state timeseries. **b)** (*left* to *right*) Run-by-run matrix of the between-run similarities, with the runs sorted in ascending order based on their free energy; free energy levels for each HMM run sorted in ascending order; between-repetition similarities of the BR-HMM approach as a function of the number of HMM runs R, from 5 to 500, in steps of 5. **c)** (*left* to *right*) Matrix of Pearson correlation coefficients between pairs of state timeseries with states ordered according to hierarchical clustering (K clusters, the total number of states is $R \times K$ =6000); dendrogram showing the states within each cluster; between-repetition similarities of the HC-HMM approach as a function of R (from 5 to 500, in steps of 5). **d)** (*left* to *right*) Overlay plots of the minimum, mean and maximum similarity scores across repetitions as a function of R (BR-HMMs in green, and HC-HMMs in orange).
